# CpG islands in *MyD88* and *ASC/PYCARD/TMS1* promoter regions are differentially methylated in head and neck squamous cell carcinoma and primary lung squamous cell carcinoma

**DOI:** 10.1186/s13000-021-01078-3

**Published:** 2021-02-26

**Authors:** Maja Šutić, Jurica Baranašić, Lana Kovač Bilić, Mario Bilić, Antonija Jakovčević, Luka Brčić, Sven Seiwerth, Marko Jakopović, Miroslav Samaržija, Ulrich Zechner, Jelena Knežević

**Affiliations:** 1 Division of Molecular Medicine, Laboratory for Advanced Genomics, Ruđer Bošković Institute, Zagreb, Croatia; 2 Department of Otorhinolaryngology, Head and Neck Surgery, Clinical Hospital Centre Zagreb, School of Medicine, University of Zagreb, Zagreb, Croatia; 3 Department of Pathology, School of Medicine, University of Zagreb, Zagreb, Croatia; 4 Diagnostic and Research Institute of Pathology, Medical University of Graz, Graz, Austria; 5 Department for Respiratory Diseases, Clinic for Respiratory Diseases Jordanovac, University of Zagreb, School of Medicine, University Hospital Centre Zagreb, Zagreb, Croatia; 6 Institute for Human Genetics, Johannes Gutenberg-University Mainz, Mainz, Germany; 7 Faculty for Dental Medicine and Health, University of Osijek, Osijek, Croatia

**Keywords:** HNSCC, Second primary tumor, Lung, Diagnostic biomarker, Methylation, CpG

## Abstract

**Background:**

Patients with head and neck squamous cell carcinoma (HNSCC) can develop lung squamous cell carcinoma (LuSCC), which could be the second primary tumor or HNSCC metastasis. Morphologically it is difficult to distinguish metastatic HNSCC from a second primary tumor which presents a significant diagnostic challenge. Differentiation of those two malignancies is important because the recommended treatments for metastatic HNSCC and primary LuSCC differ significantly. We investigated if the quantification of the promotor methylation status in HNSCC and LuSCC differs.

**Methods:**

Primary HNSCC (*N* = 36) and LuSCC (*N* = 17) were included in this study. Methylation status in the *ASC/TMS1/PYCARD* (apoptosis-associated speck-like protein containing a caspase recruitment domain; 8 CpG sites) and *MyD88* (Myeloid differentiation primary response protein 88; 10 CpG sites) promoters was analyzed. Bisulfite converted DNA, isolated from tumor tissue was quantified using pyrosequencing. Results of pyrosequencing analysis were expressed as a percentage for each tested CpG site. Receiver-operating characteristic (ROC) curve analysis was used for the evaluation of the diagnostic properties of selected biomarkers.

**Results:**

CpG sites located in the promoters of ASC/TMS1/PYCARD_CpG8 (− 65 upstream) and MyD88_CpG4 (− 278 upstream) are significantly hypermethylated in the HNSCC when compared with LuSCC (*p* ≤ 0.0001). By performing ROC curve analysis we showed that corresponding areas under the curve (AUC) were 85–95%, indicating that selected CpG sites are useful for a distinction between primary LuSCC and primary HNSCC.

**Conclusions:**

Results of the present study indicate that there is a significant difference in the methylation status of tested genes between primary HNSCC and LuSCC. However, to prove this approach as a useful tool for distinguishing second primary LuSCC from HNSCC metastasis, it would be necessary to include a larger number of samples, and most importantly, metastatic samples.

## Introduction

Patients diagnosed with head and neck squamous cell carcinoma (HNSCC) can present with lung metastases, or develop second primary lung tumors [1]. The occurrence of concurrent lung malignancies, i.e. presence of solitary lung nodules in patients with extrathoracic malignancies, like HNSCC, is frequent and always challenging for both oncologists and pathologists [2]. Even after pathologic assessment, the real nature of a solitary lung nodule in patients with HNSCC often remains ambiguous. The distinction between lung metastasis and a primary lung carcinoma for radiologists can be straightforward if metastatic disease presents with multiple lung nodules of varying sizes. On the other hand, solitary lung nodule could represent lung metastasis or primary lung carcinoma [3]. Given their morphologic similarities, lung metastases and primary squamous cell carcinoma of the lung (LuSCC) cannot be distinguished based on histopathologic characteristics. The differential diagnosis between second primary LuSCC and HNSCC metastasis in the lung is mainly dependent on clinical criteria such as localization of the lung lesion, tumor stage of HNSCC, and disease-free interval. However, some clinical criteria for this distinction, like duration of disease-free period (carcinoma in the lung is regarded as a metastasis when detected within 2–5 years after another primary tumor was diagnosed, and as a new primary when detected beyond this threshold), are empirical and non-accurate [4].

In our previous work, we have shown that the methylation status of two genes, *ASC/TMS1/PYCARD* (apoptosis-associated speck-like protein containing a caspase recruitment domain) and *MyD88* (Myeloid differentiation primary response protein 88), could be considered as a promising biomarker in non-small cell lung cancer (NSCLC) in distinguishing healthy from tumor tissue [5]. We found that both genes are hypomethylated in tumor tissue when compared to adjacent non-tumor tissue. Furthermore, we found that the methylation status of tested genes in tumor tissue could be used as a potential prognostic biomarker in patients with early-stage NSCLC because hypomethylation of specific CpG sites in *ASC/TMS1/PYCARD* gene was associated with reduced overall survival.

The fact that there is no fast and reliable diagnostic protocol for distinguishing between second primary LuSCC from HNSCC metastasis to the lung, motivated us to use primary tumor tissues (LuSCC and HNSCC) and investigate if they can be distinguished on methylation level of selected genes. We were using a pyrosequencing approach, still considered a gold standard in methylation study [6].

## Material and methods

All the protocols and molecular methods used in this study have been described in detail in our previously published data [5].

### Study cohort

This study was conducted on freshly frozen primary tumor samples. Early-stage resected squamous cell carcinoma of the lung (LuSCC) (*N* = 17) and head and neck squamous cell carcinoma (HNSCC) (*N* = 36) were obtained during surgery (University Hospital Centre Zagreb, Department for Respiratory Diseases Jordanovac, Zagreb, Croatia and Department of Otorhinolaryngology and Head and Neck Surgery, University Hospital Centre Zagreb, Croatia). Tissue samples were snap-frozen in liquid nitrogen and stored at − 80 °C. Cancer tissues were analyzed by pathologists on hematoxylin and eosin stained slides, and samples with a minimum of 70% of tumor cells were preceded for further analysis. The clinical and pathological features of the patients are shown in Table 1. All patients signed informed consent and the study was approved by the local ethics committee (University Hospital Centre Zagreb).

**Table 1:** Clinical characteristics of the study cohort. Data are presented as number (%) unless otherwise indicated

Characteristic	HNSCC N (%)	LuSCC N (%)
Age at diagnosis, years, median (range)	62 (40–80)	63 (38–79)
Number of patients	36 (100)	17 (100)
T-stage		
1	0	0
2	15 (41.7)	12 (70.6)
3	12 (33.3)	4 (23.5)
4	9 (25.0)	1 (5.9)
N-stage		
0	23 (63.9)	9 (52.9)
1	2 (5,6)	7 (41.2)
2	11 (30.5)	1 (5.9)
TNM-stage		
1	0	3 (17.6)
2	14 (38.9)	11 (64.7)
3	9 (25)	3 (17.6)
4	13 (36.1)	0

### CpG analysis

**Fig. 1:**
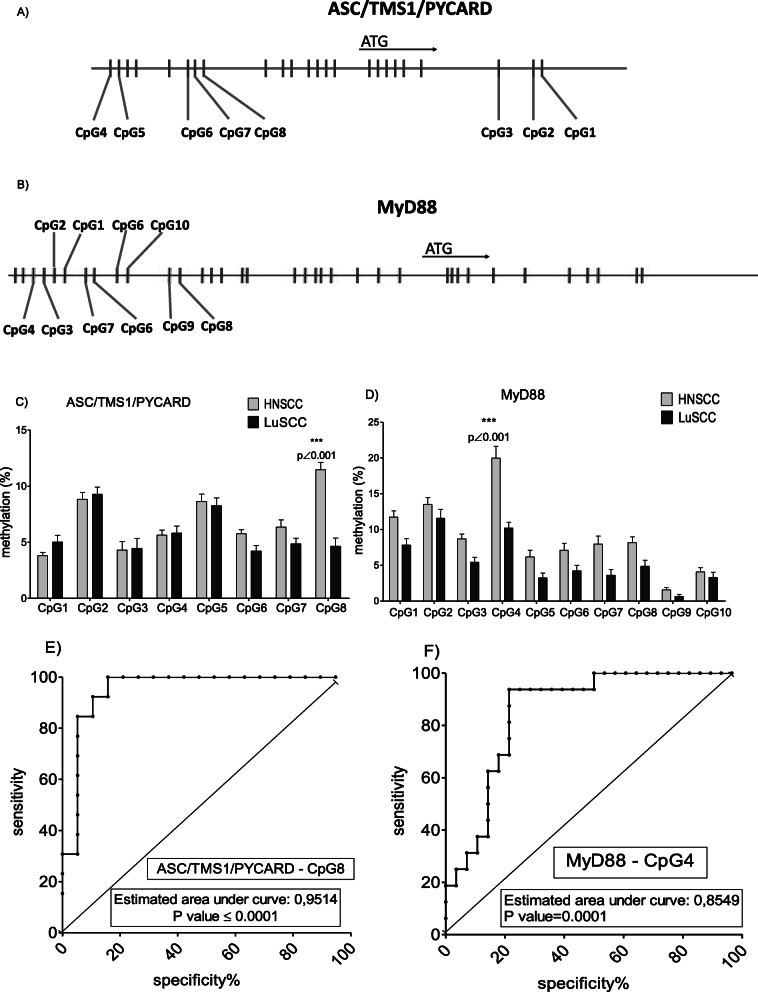
Position of the tested CpG sites in the *ASC/TMS1/PYCARD* (**a**) and *MyD88* (**b**) gene promoters (vertical lines)**.** Methylation status of selected CpC sites in the promoter region of the tested genes (**c** and **d**); each bar represents mean percentage methylation (%) of individually tested CpG site; methylation status of all tested CpGs in all tested samples are represented as an average value (%) of at least 3 individual PCR/pyrosequencing reactions. ROC curve analysis for *ASC/TMS1/PYCARD* CpG8 (**e**) and *MyD88* CpG4 (**f**) for differentiation of primary HNSCC from primary LuSCC

DNA, isolated from freshly frozen tumor tissue samples, was bisulfite converted, amplified, and sequenced. For this study, we have analyzed the methylation status of the *MyD88* and *ASC/TMS1/PYCARD* genes in the primary HNSCC and compared them with methylation status in the primary LuSCC samples obtained in the previously mentioned study. We have analyzed 8 different CpG sites in the promoter region of the *ASC/TMS1/PYCARD* gene and 10 different CpG sites in the *MyD88* gene. Locations of the tested CpG sites in the *ASC/TMS1/PYCARD* gene are as follows: CpG1 + 52; CpG2 + 49; CpG3 + 33; CpG4–134; CpG5–129; CpG6–76; CpG7–71; CpG8–65. Locations of the tested CpG sites in the *MyD88* gene are as follows: CpG1–253; CpG2–256; CpG3–267; CpG4–278; CpG5–210; CpG6–216; CpG7–222; CpG8–146; CpG9–151; CpG10–167. Indicated values represent a distance from the transcriptional start site. Schematic locations of the tested CpG sites and tested genes are presented in the Fig. 1a and b. Results of the pyrosequencing analysis were expressed as a percentage for each analyzed CpG site.

### Statistical analysis

Differences in methylation status were tested using a paired t-test. To define the possible diagnostic applicability of selected CpG sites, receiver operating characteristic (ROC) curves analysis was performed. To assess the diagnostic properties of selected CpG sites, ROC curves, the areas under curves (AUC) together with 95% confidence intervals (95% CI) were plotted. For this analysis values of methylation status (%) of selected CpG sites (*ASC/TMS1/PYCARD*_CpG8 and *MyD88*_CpG4), detected for each sample in HNSCC and LuSCC, were plotted.

## Results

Analysis of the methylation status demonstrated that the methylation pattern of *ASC/TMS1/PYCARD* and *MyD88* promoters is different between primary tumors in HNSCC and LuSCC sample cohorts. We have shown that *ASC/TMS1/PYCARD*_CpG8 (located − 65 upstream) is significantly hypermethylated in primary HNSCC (11.47%) in comparison to LuSCC (4.65%) (*p* < 0.0001) (Fig. 1c). Additionally, we have found that *MyD88*_CpG4 (located − 278 upstream) is significantly hypermethylated in HNSCC (19.96%) when compared to LuSCC (10.2%) (p < 0.0001) (Fig. 1d). Results of ROC curve and AUC analysis indicated that both CpG sites are promising biomarker candidates because corresponding AUC were ≥ 0.85 (**Fig.** 1e and f), showing that selected markers could potentially have clinical utility.

## Discussion

Results of our study demonstrated a difference in methylation of CpG islands in *MyD88* and *ASC/PYCARD/TMS1* promoter regions in primary tumors of HNSCC and LuSCC. As already mentioned, the distinction between metastatic HNSCC from the second primary LuSCC is difficult but crucial for prognosis and treatment options. The second primary lung carcinoma may be low stage and resectable, whereas a metastasis of an HNSCC is a clear sign of tumor dissemination and disease progression, typically without options for curative intervention. A histopathologic distinction is not reliable, and in most cases, not possible. Therefore, development of the novel diagnostic strategies based on precise molecular genetic markers and their implementation is of great interest. It has been observed that specific epigenetic changes, especially DNA methylation, have potential as diagnostic biomarkers. To the best of our knowledge, there is only one study analysing DNA methylation profiling of primary HNSCC and LuSCC which results were further used for different machine learning methods to distinguish metastatic HNSCC from second primary LuSCC [7]. They have demonstrated that DNA methylation profiling is superior in differentiating lung metastases of HNSCC from primary lung cancers arising in patients with HNSCC and, in conjunction with machine learning methods, can solve this specific diagnostic problem.

Our study has several limitations. First of all the study cohort is rather small, therefore we regard our study as a pilot study, whose results need to be reconfirmed by independent studies in different cohorts. Second, due to the small cohort size, clinical/pathological characteristics of patients included are unbalanced, which might also influence our results. Here, we need to stress that we have performed a comparison of obtained methylation data in early-stage HNSCC with early-stage LuSCC (data not presented), and the results were the same as presented for the whole cohort. The third, and probably the most important limitation, is that we did not compare lung metastases of HNSCC with primary LuSCC and primary HNSCC. To further define tested loci as potential diagnostic markers, this is necessary.

In conclusion, the methylation status of selected genes indicated that it is different in primary tumor tissues of different origins. Further analyses, on a larger number of the samples including metastasis, are needed to confirm its utility for a distinction between second primary LuSCC and metastatic HNSCC in the lung. Results of this pilot study must be considered as preliminary and additional independent studies are necessary to confirm the diagnostic utility of the tested loci/approach.

## Data Availability

The datasets used and/or analyzed during the current study are available from the corresponding author on reasonable request.

## Abbreviations

HNSCC: Head and neck squamous cell carcinoma
LuSCC: Lung squamous cell carcinoma
*MyD88*: Myeloid differentiation primary response protein 88
*ASC/TMS1/PYCARD*: Apoptosis-associated speck-like protein containing a caspase recruitment domain
NSCLC: Non-small cell lung cancer
ROC: Receiver operating characteristic
AUC: Area under curve
